# Correction: Tim-3 Expression in Cervical Cancer Promotes Tumor Metastasis

**DOI:** 10.1371/journal.pone.0152830

**Published:** 2016-03-29

**Authors:** Yang Cao, Xiaoxi Zhou, Xiaoyuan Huang, Qinlu Li, Lili Gao, Lijun Jiang, Mei Huang, Jianfeng Zhou

The authors would like to correct Fig 5, as errors were introduced in the preparation of this figure for publication. In Fig 5D, the assay image for ADV-GFP is derived from the same microscopic image as the assay of PBS. The authors have provided a corrected version of Fig 5 here.

**Fig 5:**
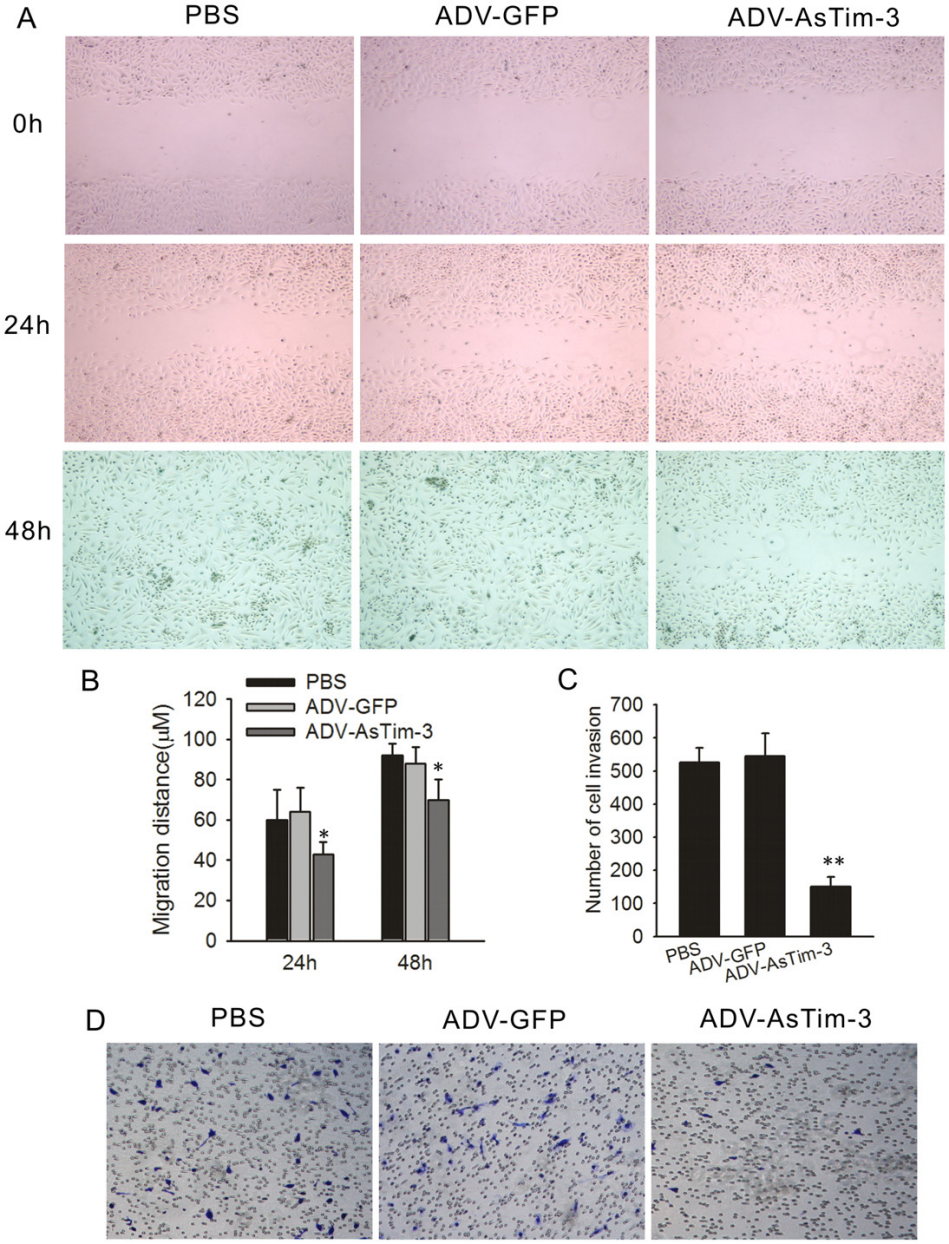
Effect of Tim-3 inhibition on Hela cell migration and invasion in vitro. (A) Cell migration capability was determined with a wound healing assay. Photographs were taken immediately (0 h), at 24 h and 48 h after wounding. (B) Quantification of wound closure. The data present the mean distance of cell migration to the wound area at 24 h and 48 h after wounding in three independent wound sites per group. (C) The ability of the cells to invade Matrigel was analyzed by the transwell invasion assay through a gel matrix. Hela cells were either infected with ADV-GFP or with ADV-antisense Tim-3, After 10 h viable invasive cells were fixed and counted. Values and error bars shown in this graph represent the averages and standard deviations respectively, of three independent experiments. (D) Representative images of the transwell invasion assay.

The authors confirm that these changes do not alter their findings. The authors have provided the underlying images for all figures in the original article as Supporting Information.

## Supporting Information

S1 File Underlying images for all figures. (ZIP)
