# Supplementary figures and images for: Correction: Tim-3 Expression in Cervical Cancer Promotes Tumor Metastasis
Source: PLoS One. 2016 Mar 29;11(3):e0152830. doi: 10.1371/journal.pone.0152830 (PMC4811421; doi:10.1371/journal.pone.0152830)

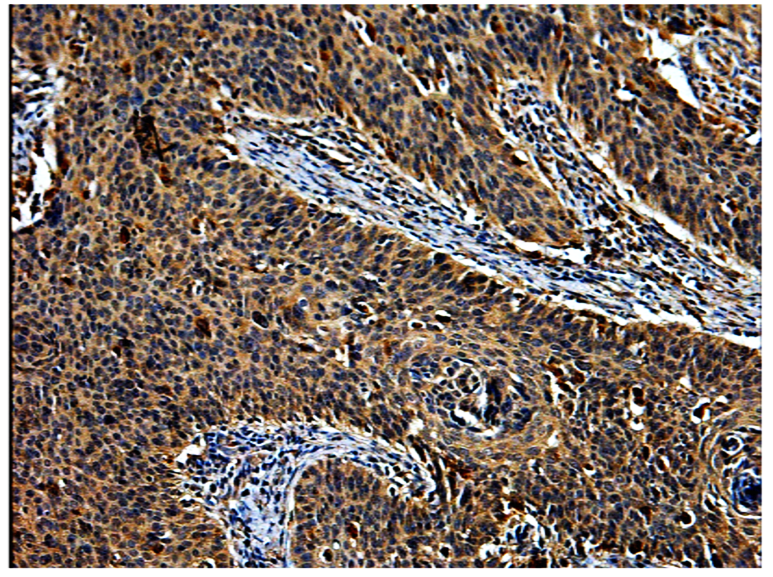

Supplement: S1 File — (ZIP) [file pone.0152830.s001.zip › Fig 1/Figure 1A.tif]

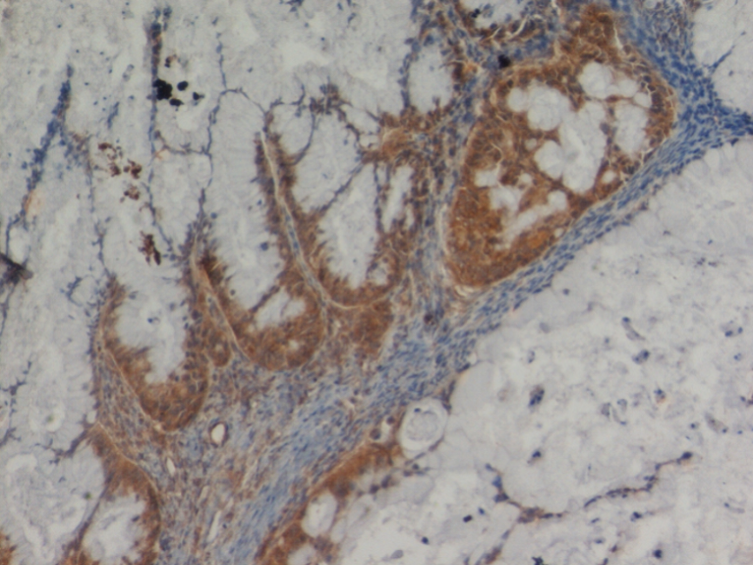

Supplement: S1 File — (ZIP) [file pone.0152830.s001.zip › Fig 1/Figure 1B.tif]

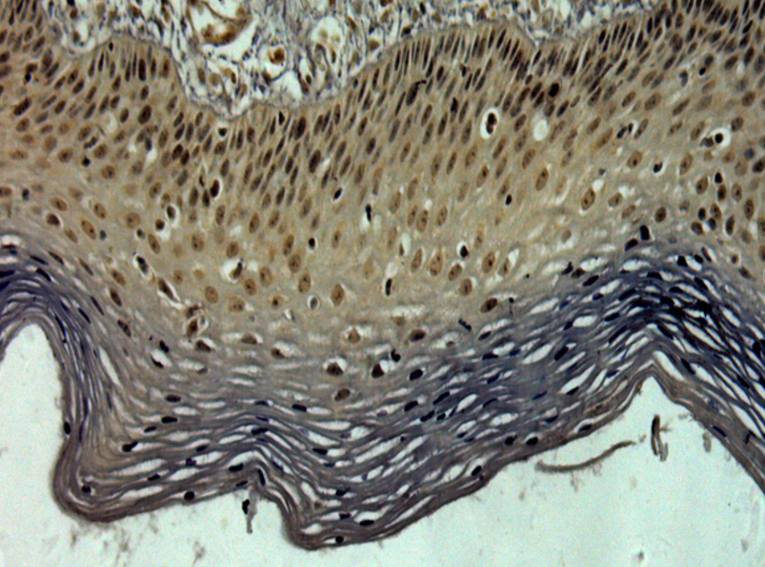

Supplement: S1 File — (ZIP) [file pone.0152830.s001.zip › Fig 1/Figure 1C.tif]

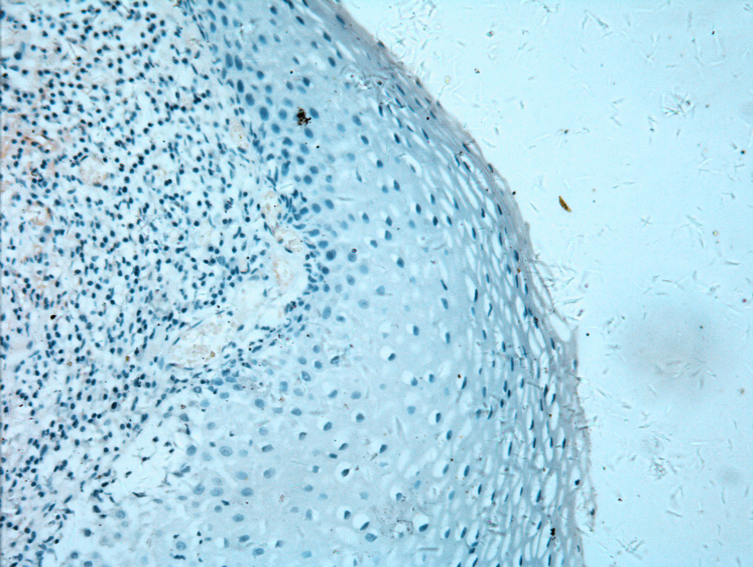

Supplement: S1 File — (ZIP) [file pone.0152830.s001.zip › Fig 1/Figure 1D.tif]

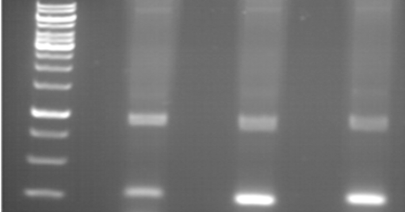

Supplement: S1 File — (ZIP) [file pone.0152830.s001.zip › Fig 3/Figure 3A.tif]

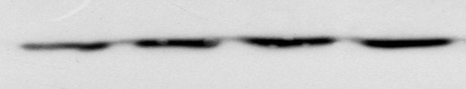

Supplement: S1 File — (ZIP) [file pone.0152830.s001.zip › Fig 3/Figure 3B lower panel.tif]

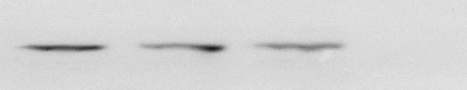

Supplement: S1 File — (ZIP) [file pone.0152830.s001.zip › Fig 3/Figure 3B upper panel.tif]

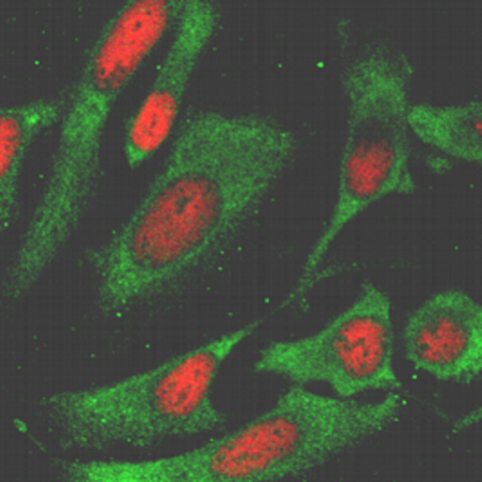

Supplement: S1 File — (ZIP) [file pone.0152830.s001.zip › Fig 3/Figure 3C left panel.tif]

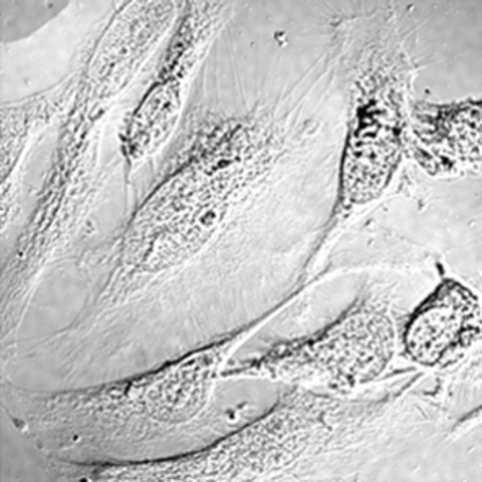

Supplement: S1 File — (ZIP) [file pone.0152830.s001.zip › Fig 3/Figure 3C right panel.tif]

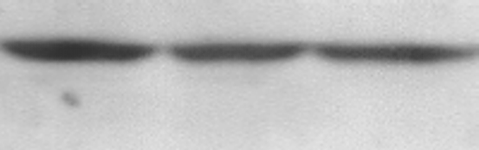

Supplement: S1 File — (ZIP) [file pone.0152830.s001.zip › Fig 4/Figure 4A lower panel.tif]

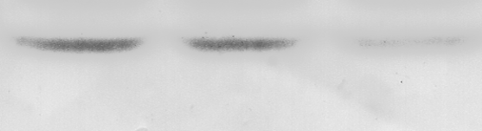

Supplement: S1 File — (ZIP) [file pone.0152830.s001.zip › Fig 4/Figure 4A upper panel.tif]

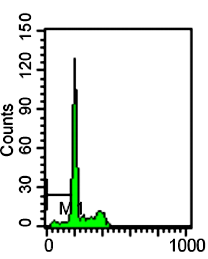

Supplement: S1 File — (ZIP) [file pone.0152830.s001.zip › Fig 4/Figure 4B middle panel.tif]

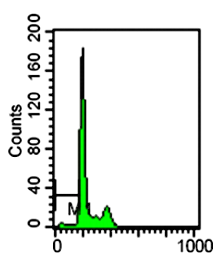

Supplement: S1 File — (ZIP) [file pone.0152830.s001.zip › Fig 4/Figure 4B right panel.tif]

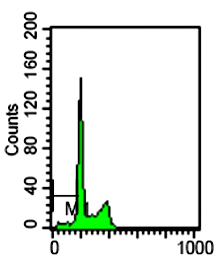

Supplement: S1 File — (ZIP) [file pone.0152830.s001.zip › Fig 4/Figure 4C left panel.tif]

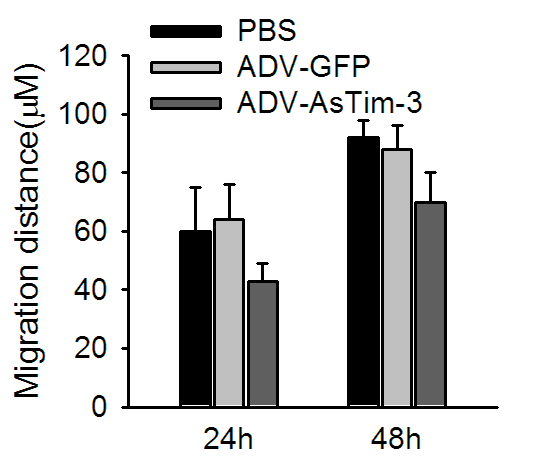

Supplement: S1 File — (ZIP) [file pone.0152830.s001.zip › Fig 5/Figure 5B.TIF]

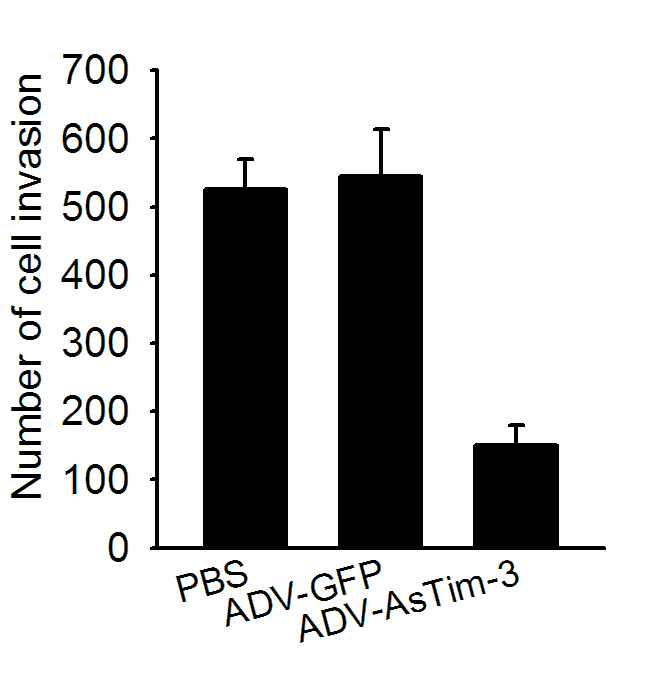

Supplement: S1 File — (ZIP) [file pone.0152830.s001.zip › Fig 5/Figure 5C.TIF]

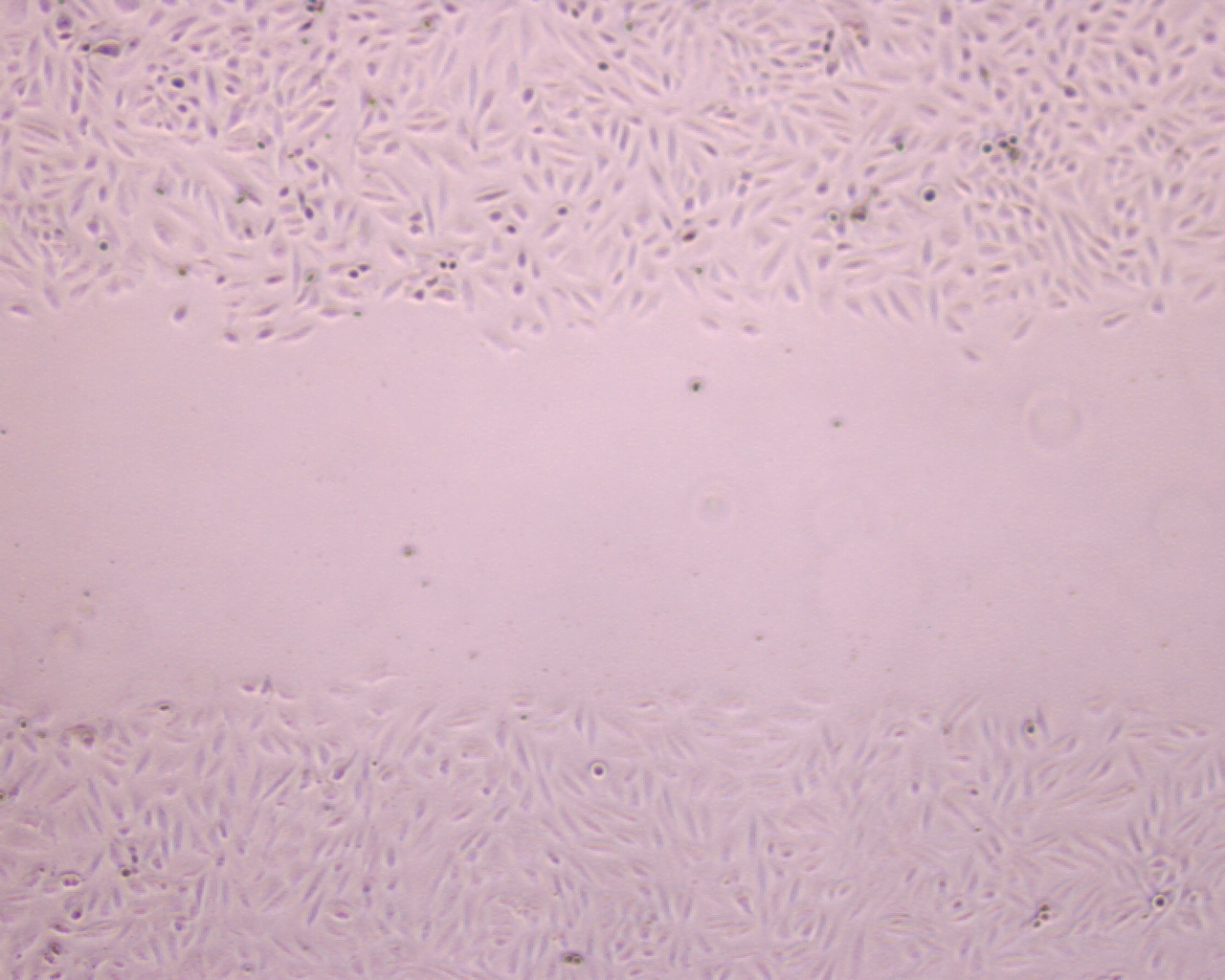

Supplement: S1 File — (ZIP) [file pone.0152830.s001.zip › Fig 5/Panel A/Figure 1.jpg]

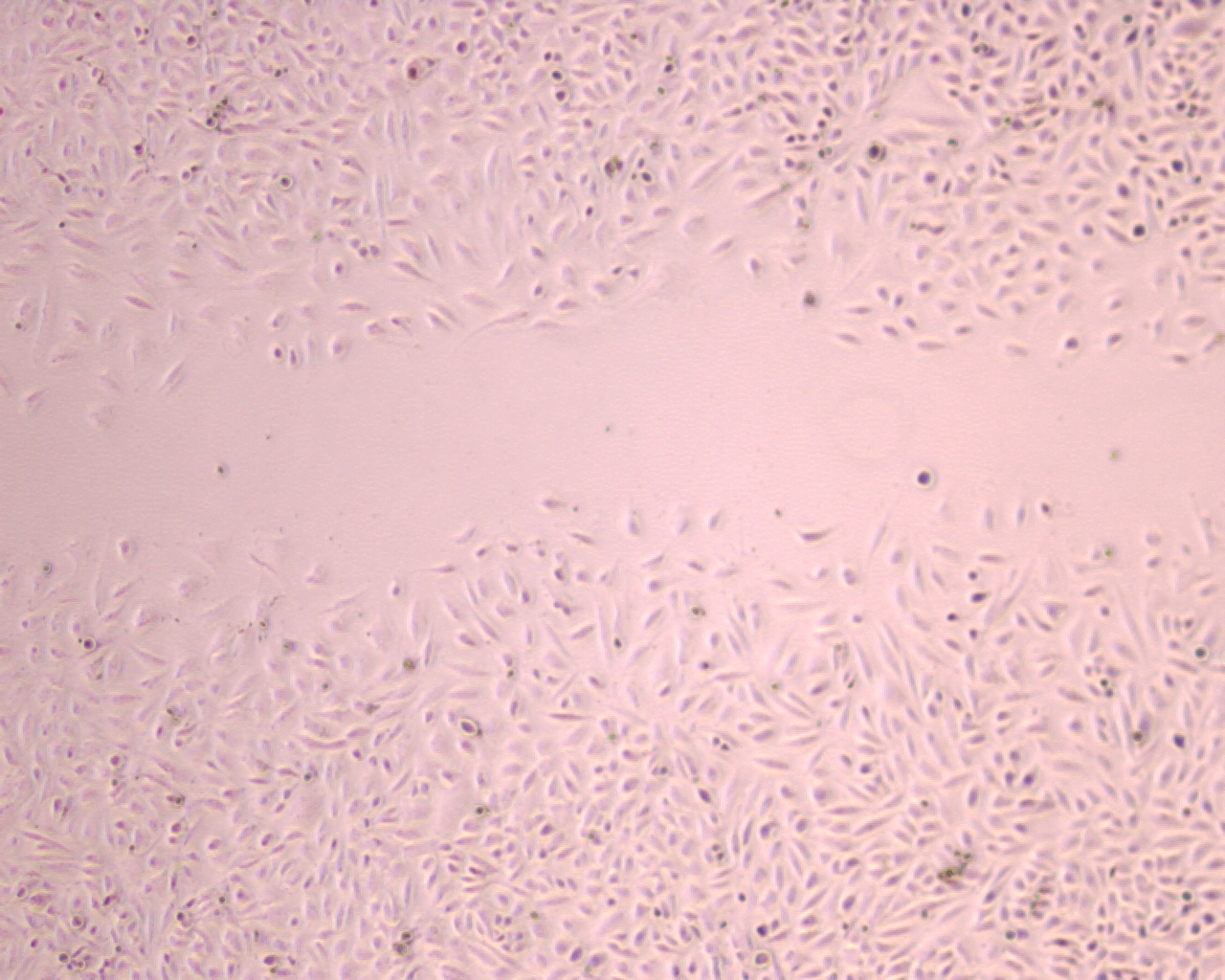

Supplement: S1 File — (ZIP) [file pone.0152830.s001.zip › Fig 5/Panel A/Figure 2.jpg]

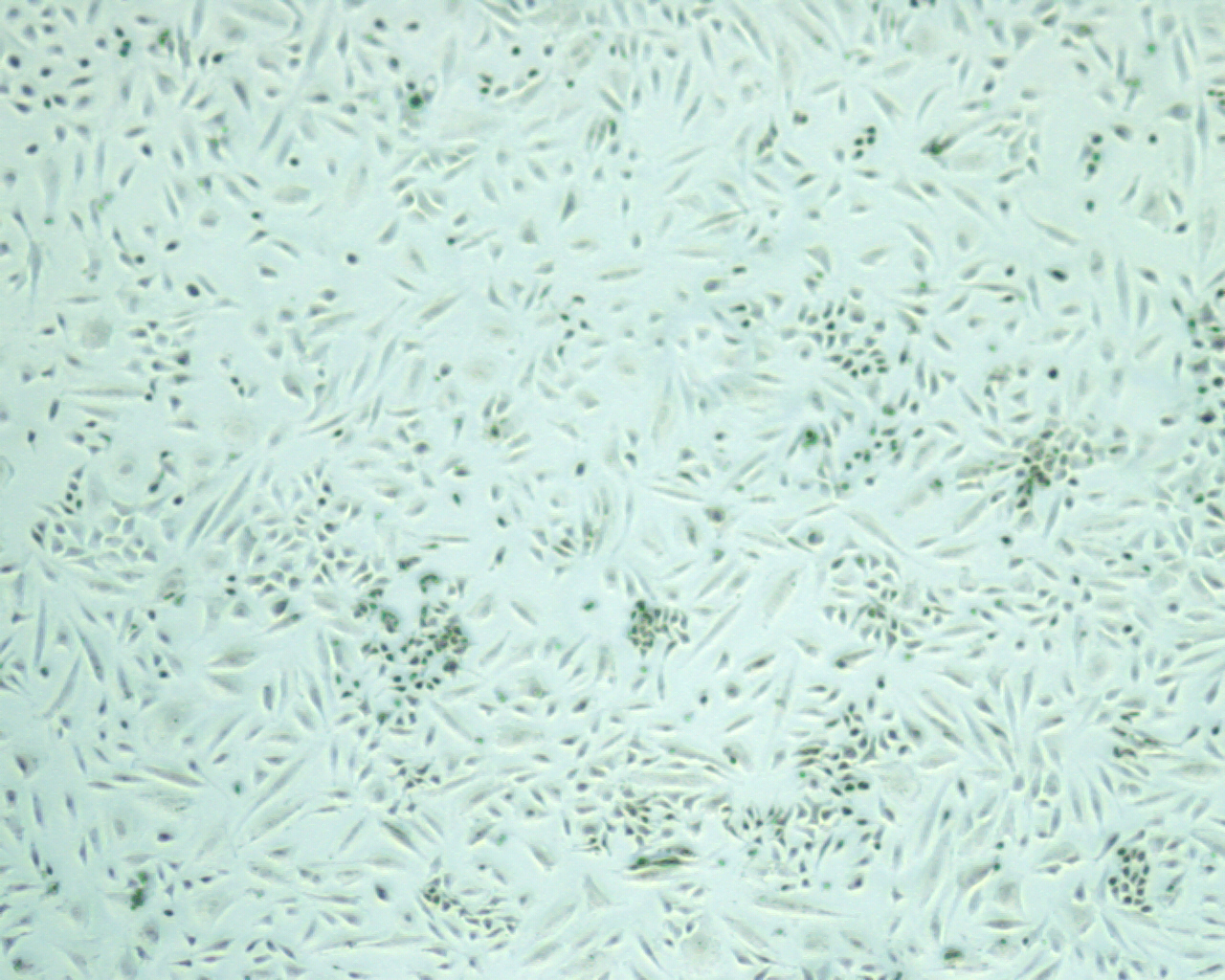

Supplement: S1 File — (ZIP) [file pone.0152830.s001.zip › Fig 5/Panel A/Figure 3.jpg]

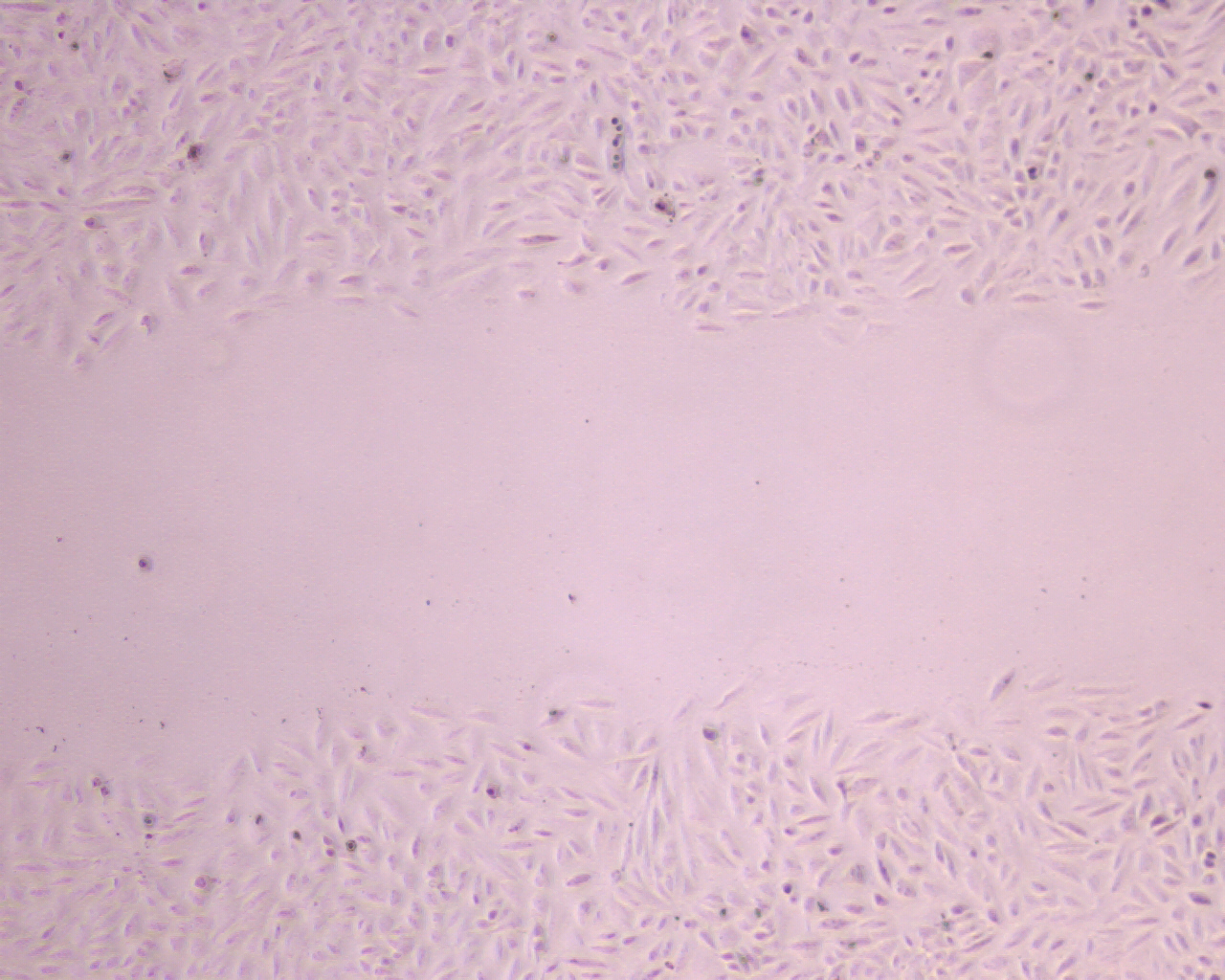

Supplement: S1 File — (ZIP) [file pone.0152830.s001.zip › Fig 5/Panel A/Figure 4.jpg]

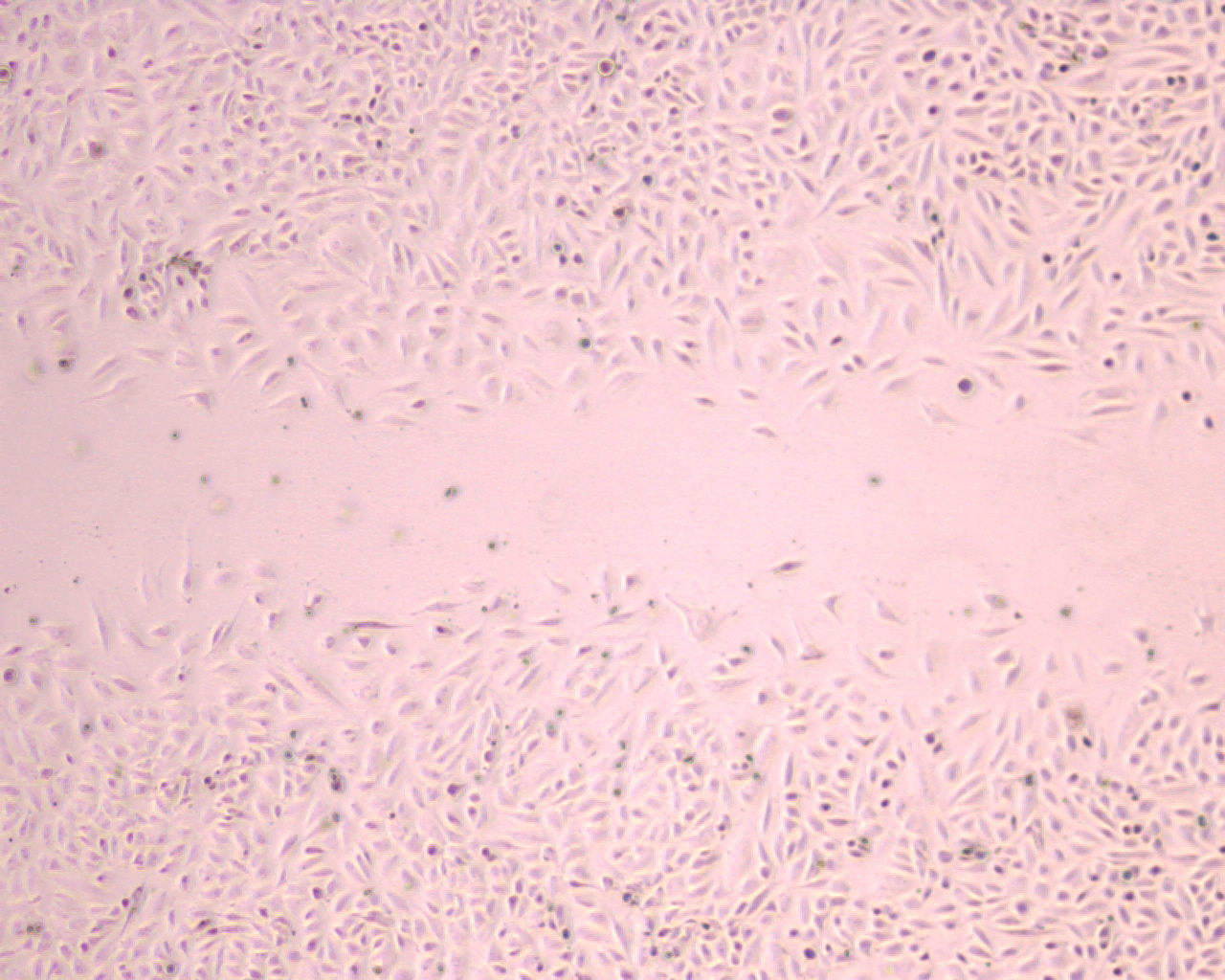

Supplement: S1 File — (ZIP) [file pone.0152830.s001.zip › Fig 5/Panel A/Figure 5.jpg]

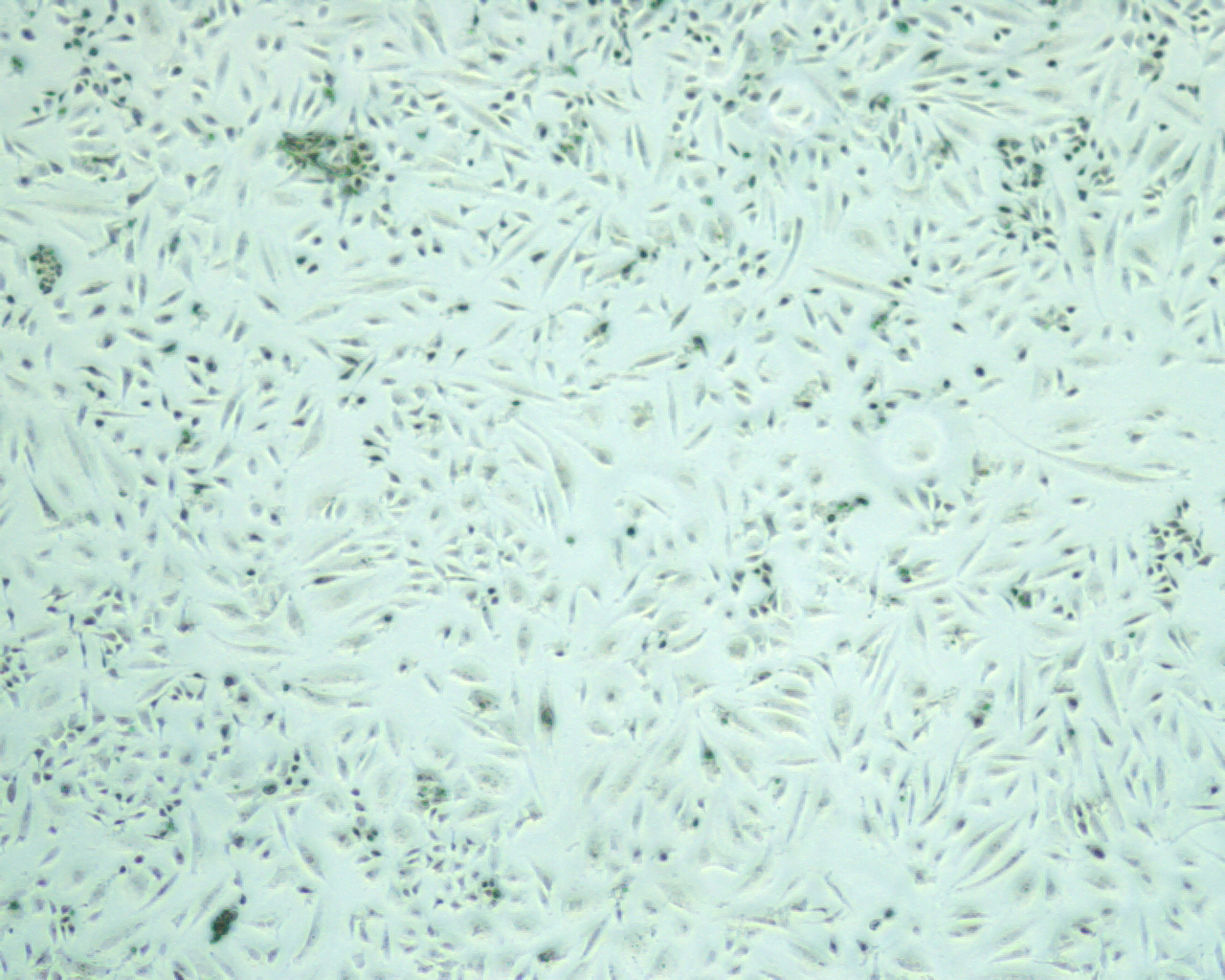

Supplement: S1 File — (ZIP) [file pone.0152830.s001.zip › Fig 5/Panel A/Figure 6.jpg]

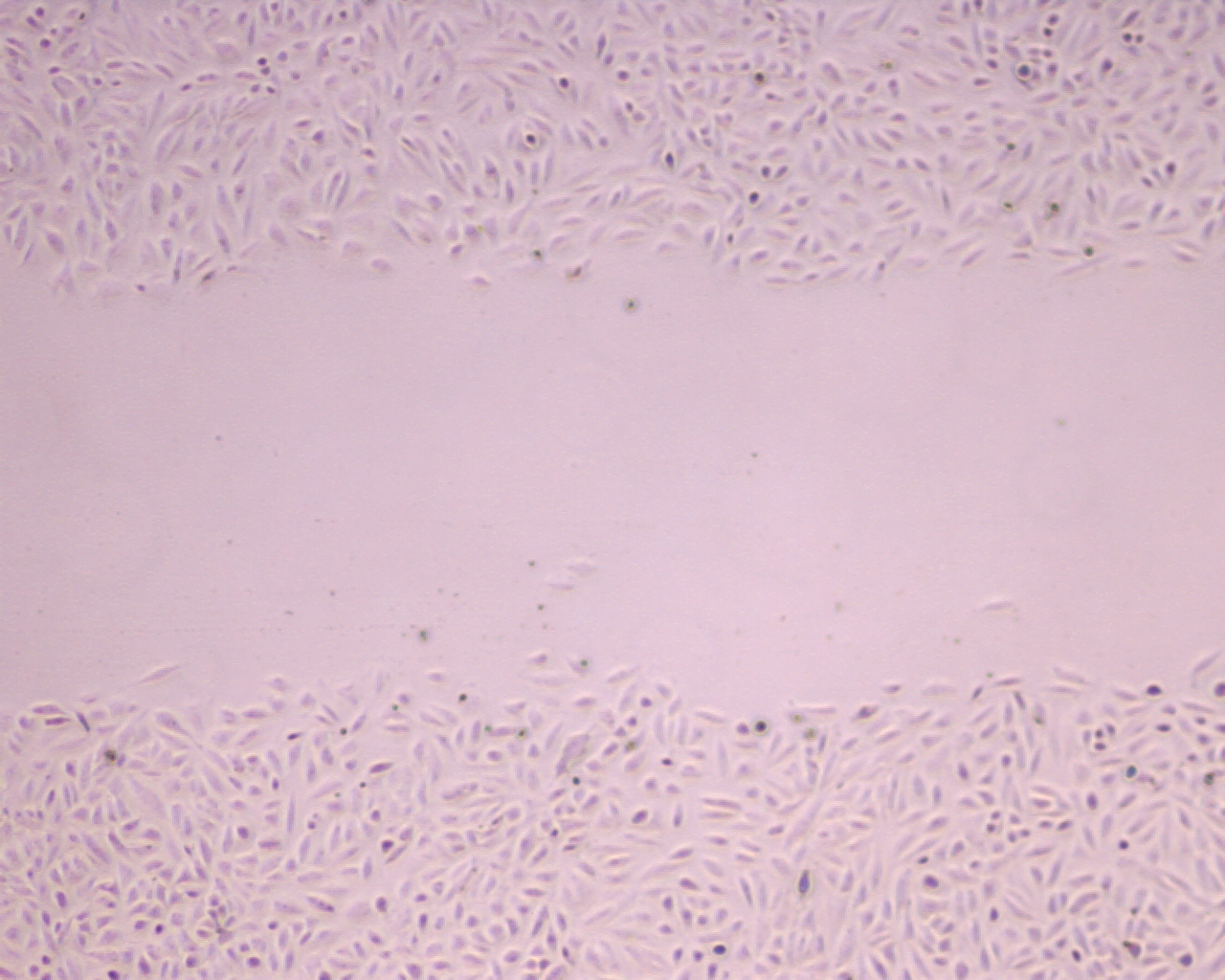

Supplement: S1 File — (ZIP) [file pone.0152830.s001.zip › Fig 5/Panel A/Figure 7.jpg]

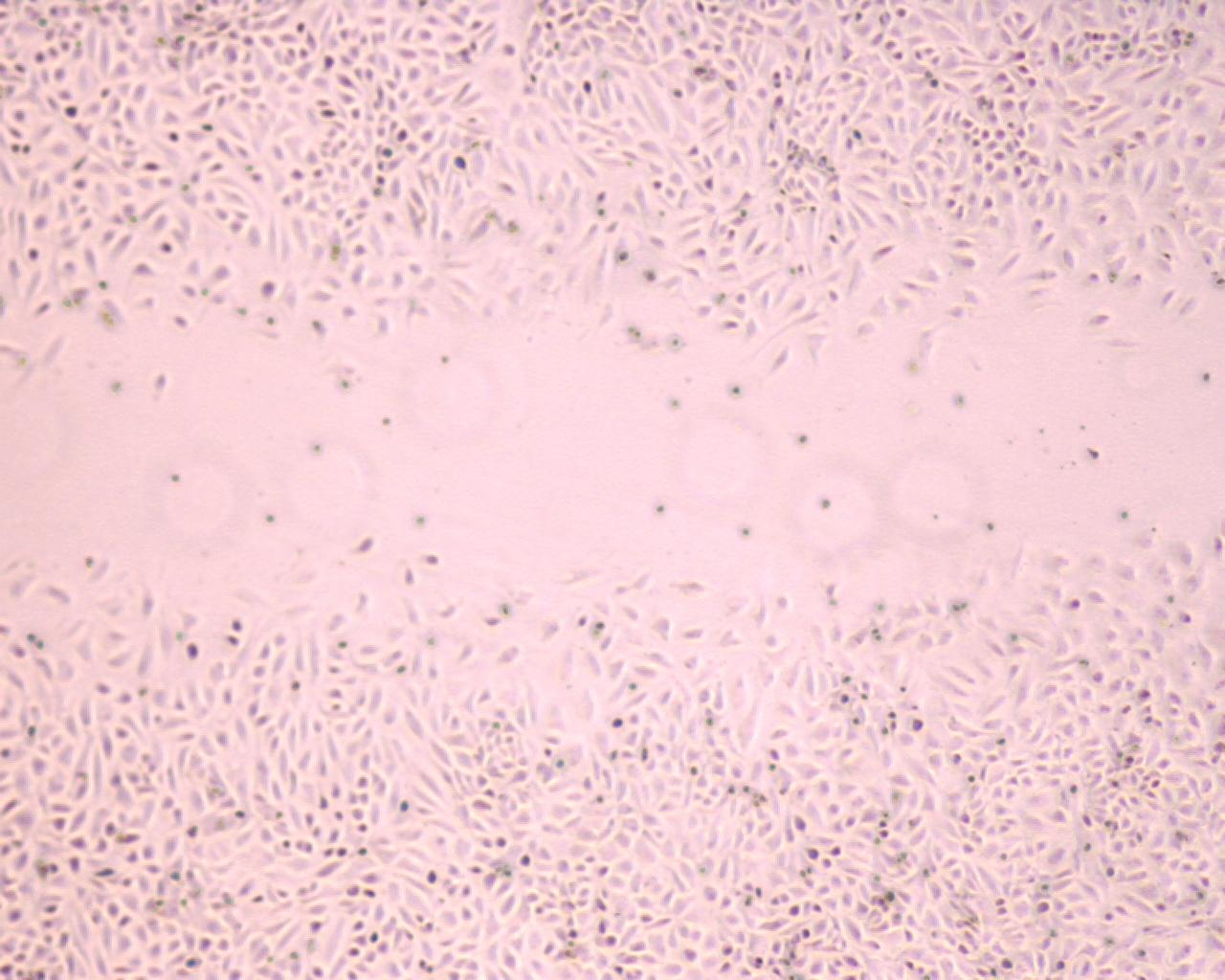

Supplement: S1 File — (ZIP) [file pone.0152830.s001.zip › Fig 5/Panel A/Figure 8.jpg]

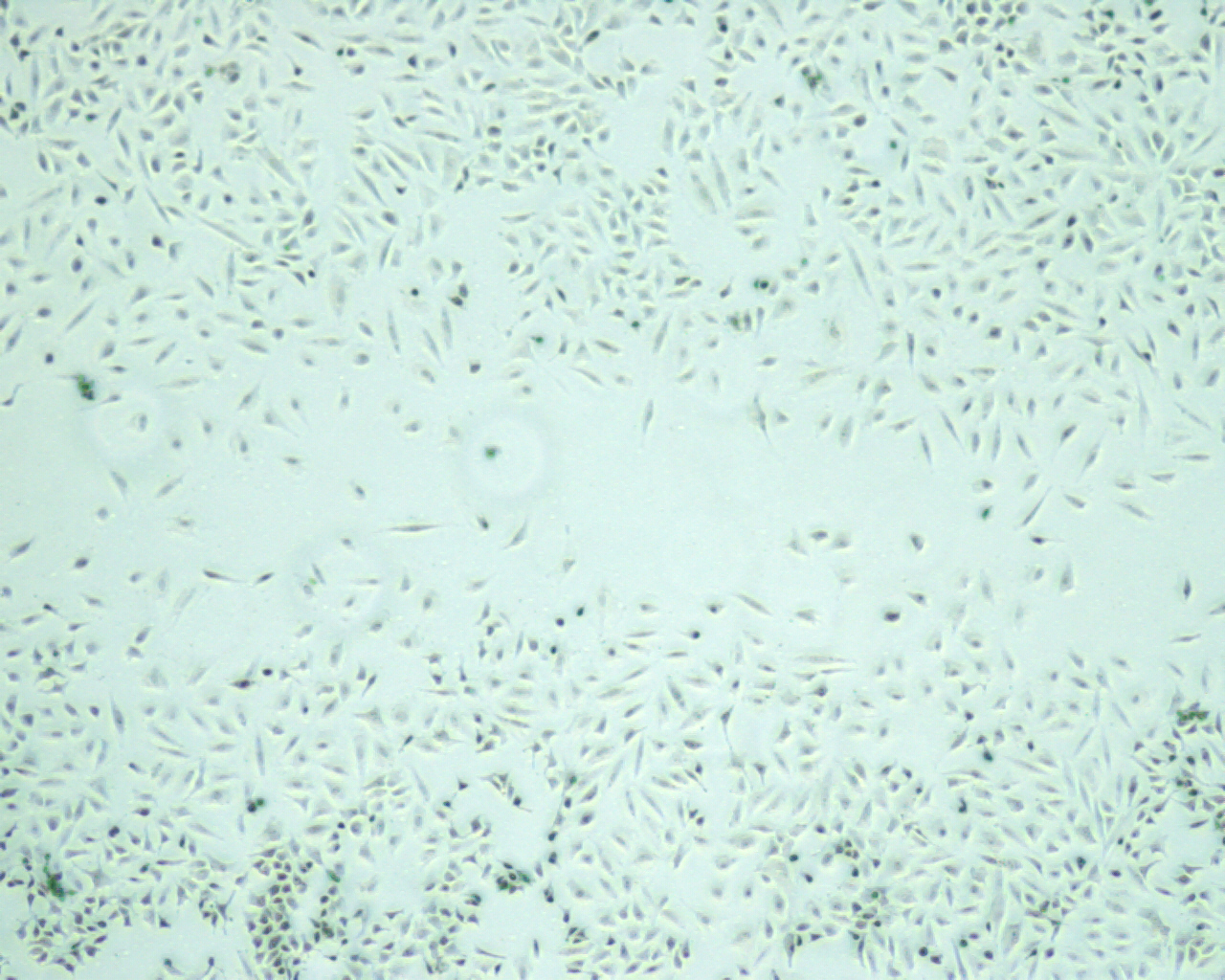

Supplement: S1 File — (ZIP) [file pone.0152830.s001.zip › Fig 5/Panel A/Figure 9.jpg]

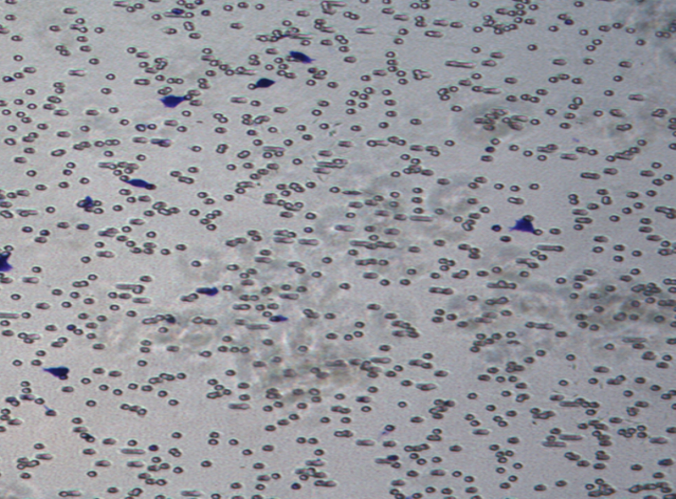

Supplement: S1 File — (ZIP) [file pone.0152830.s001.zip › Fig 5/Panel D/ADV-AsTim-3.tif]

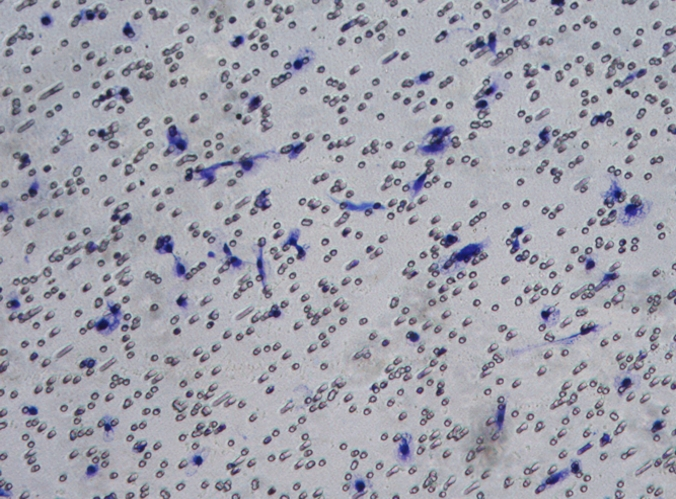

Supplement: S1 File — (ZIP) [file pone.0152830.s001.zip › Fig 5/Panel D/ADV-GFP.tif]

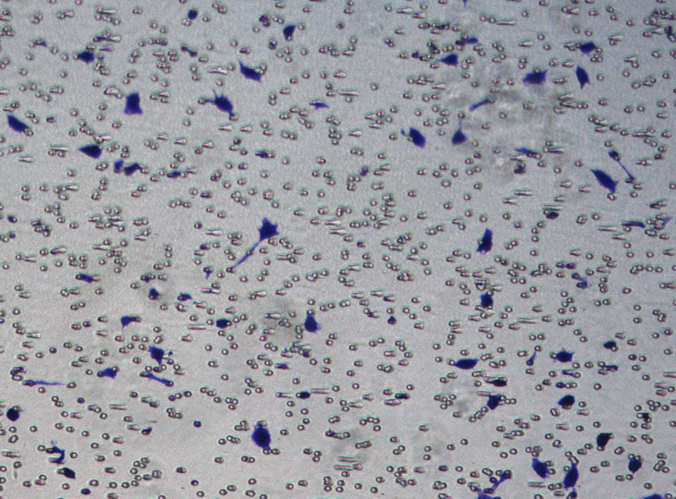

Supplement: S1 File — (ZIP) [file pone.0152830.s001.zip › Fig 5/Panel D/PBS.tif]
